# Citizens' Jury as a Strategy to Increase Social Participation Among Older People

**DOI:** 10.1111/hex.70424

**Published:** 2025-09-10

**Authors:** Nancy Flores‐Castillo, Carmen García‐Peña, Sara Torres‐Castro, Héctor García‐Hernández

**Affiliations:** ^1^ National Institute of Geriatrics Mexico City Mexico

**Keywords:** citizen jury, democratic deliberation, older people, research priority, social participation, translational science

## Abstract

**Introduction:**

Social participation is essential for healthy ageing. Older people must engage in social, political and cultural activities. Institutions must create spaces that enable their involvement. Citizens' jury (CJ) is a strategy that promotes participation by involving citizens in deliberative processes. We organised a CJ with older people at the National Institute of Geriatrics (INGER) to identify ageing‐related research priorities for the institute.

**Methods:**

We conducted a CJ of older people. While there's no standard procedure for performing it, four elements are central: the jury, experts, facilitators and observers. Four researchers from INGER—representing basic, clinical, socio‐demographic and technological research areas—served as expert presenters. The CJ was held over 2 days in January 2025. Jurors were asked: What are the priority ageing research topics that should be investigated at the INGER?

**Results:**

The jury included 27 people, mainly aged 60–69, most of them women, and 74% had at least a bachelor's degree. The basic research area received the least attention, while the clinical and social areas drew more interest. Technology also emerged as a relevant area. Jurors highlighted overlooked topics such as emotional and sexual health, which are rarely investigated within the institute. They also stressed the need for better dissemination, prevention‐oriented research, intersectoral collaboration, and the active inclusion of older people in the research process. The jury called for a more comprehensive, life‐course and intersectional research perspective.

**Conclusions:**

The CJ is a deliberative strategy that can be used to foster social participation among older people. It enables their active involvement in diverse public issues, such as the identification of research priorities. Additionally, we enhance knowledge translation by promoting research agendas with topics that matter to this age group.

**Public Contribution:**

Jury participants were the central element that made this strategy possible. They received enough information from experts on the different ageing research areas and actively engaged in deliberation. Their role went beyond giving opinions; they participated in the identification and prioritisation of research topics.

## 1 Introduction

The World Health Organization (WHO) has emphasised that social participation is one of the key components of healthy ageing [1]. Social participation refers to the active engagement of individuals in formal and non‐formal community organisations, necessarily requiring interpersonal interaction [2]. These interactions promote socialisation, self‐realisation, strengthened interpersonal relationships, and the achievement of personal goals [1]. It is considered a fundamental element of the age‐friendly cities strategy [3], and research has demonstrated its association with maintaining functionality [4], reducing chronic morbidity [5], improving mental health [2] and lowering mortality rates [6].

Various political initiatives, such as the International Covenant on Economic, Social and Cultural Rights [7], the Madrid International Plan of Action on Ageing [8], the Inter‐American Convention on Protecting the Human Rights of Older people [9], the 2030 Agenda for Sustainable Development [10], and the Decade of Healthy Ageing [11], have indicated that older people ought to stay engaged in the community and that they have the right to participate in social, political, economic, cultural and spiritual activities without facing age‐based discrimination. These initiatives also emphasise that societies should actively promote their participation [11, 12, 13].

However, older people are a population group that may face social exclusion due to a complex interplay of factors, including reduced mobility, urban infrastructure barriers, limited access to employment opportunities, discrimination within social institutions, and a societal emphasis on youth that undervalues their contributions [13, 14]. Consequently, since the late 20th century, efforts have been made to empower older citizens and actively involve them in social activities [12, 13, 14, 15]. Nevertheless, their engagement is strongly influenced by societal ageism and the availability of institutional opportunities. Therefore, fostering participation opportunities within institutions is essential to promoting the social involvement of older people [15].

Citizens' jury (CJ) is one of the most used deliberative techniques in public health to promote social participation [16]. It allows significant public involvement and facilitates bidirectional communication between citizens, researchers and decision‐makers [17]. A CJ is a public participation technique where a representative group from the community receives enough information about a specific topic within a short time frame. This enables them to understand and discuss complex and contentious issues, ultimately producing a final opinion that considers the perspectives of all jury participants [18, 19, 20]. Utilising this approach ensures that citizens' perspectives are acknowledged and incorporated into decision‐making processes [21].

CJs have been used in non‐older adult groups to address various health issues, including discussions on regulatory responses to health‐promoting measures against McDonald's food [22], strategies for improving school immunisation programs [23], public perspectives on private data management in health research [24], and public priorities for government expenditure on obesity surgical management [25]. In Australia and New Zealand, CJ has been used to identify priority research topics for incarcerated populations [26] and women living with urinary incontinence [27], respectively.

Given the importance of promoting social participation in this age group, we conducted a CJ with older Mexican people at the National Institute of Geriatrics (INGER) to engage them through this participatory approach aimed at identifying research priorities on ageing within the Institute.

## 2 Materials and Methods

We conducted a CJ of older people. The team posted calls on INGER social networks in November–December 2024. We considered the following inclusion criteria: being 60 years or older, living in Mexico City, being able to travel to INGER independently, and having at least 6 hours available for a 2‐day meeting. The only exclusion criterion was having a consanguineous relationship with the organisers of the jury. Calls for participation included a registration link, where interested individuals provided their phone number and an electronic mail address. Afterwards, participants were contacted by telephone, and an information sheet was sent via WhatsApp, explaining what a CJ was, the purpose of the jury, and recommendations for participation.

We received a total of 65 respondents. After excluding those who did not fulfil the inclusion criteria, the sample was integrated by 27 older people. Two weeks before the CJ, all participant members receive logistical details, the meeting agenda, an informed consent form, a letter for the use of personal image, and introductory materials on the four types of scientific research conducted at INGER. Basic research refers to studies that seek to understand the genetic, molecular and biological processes involved in ageing. Clinical research focuses on how to prevent, diagnose and treat diseases in older people. Socio‐demographic research analyses the impact of ageing on society, particularly in areas such as health, the economy and public policy. Lastly, gero‐technological research aims to develop and evaluate tech innovations that can improve the quality of life of older people.

Jury members were encouraged to actively participate and answer questions about each presentation. Also, we sent a socio‐demographic questionnaire, which includes questions about age, sex, level of education, marital status, social security affiliation, chronic diseases and disability, the latter measured using the Washington Group consensus [28].

### 2.1 CJ Process

There is no standard procedure for performing a CJ. However, four key elements must be considered: (1) the jury itself, (2) the ‘experts’, (3) the facilitators, and (4) the ‘watchers’ [16, 17, 19]. The ‘experts’ provide neutral and unbiased information to the jury about the different aspects of the issue. Also, they answer all the jury members' doubts, questions, and concerns [19]. The facilitators support the jurors in navigating a well‐organised conversation to form a collective opinion within the set time limits. Additionally, they coordinated the expert presentations, ensured the discussions ran smoothly, kept track of time, and directed attention to the core issues [22]. On the other hand, the ‘watchers’ guarantee the neutrality of the process. They also register the main points of the discussion. In this CJ, four experts, two facilitators, and one watcher were considered.

Experts comprise researchers with a solid academic trajectory, one from each of the four areas or fields of research conducted at INGER. Personalised invitations were sent, and they were invited to a preparatory meeting in November 2024, where the research team presented the project and outlined its objective. Additionally, they were briefed on the methodology to be used and the meeting structure. They were also advised to maintain neutrality throughout the meeting to prevent undue influence on the deliberation process. The research expert's presentation followed a structured format, including a definition of the research field, main objectives, examples of ageing research at international and national levels, and a research project conducted at INGER.

For the deliberative work of the CJ, we followed the guidelines of other previous experiences [17, 29]. The CJ meeting took place over 2 days in January 2025. Although the jury was scheduled to meet from 9:00 to 14:00 hours, the group arrived an hour early on both days of work, meaning the work for both sessions lasted a total of 12 hours. During the sessions, the jury was asked to respond: What are the priority ageing research topics that should be investigated at the INGER? On Day 1, the jury members signed the informed consent and letter for the use of their personal images. Also, they established democratically the working conditions during the meeting, which included agreements such as maintaining confidentiality, participating actively, communicating clearly, and addressing others with respect. These agreements facilitated the deliberative process and allowed for greater group integration. Then, an introductory technique was used to raise awareness and encourage reflection on the importance of scientific research in ageing. Subsequently, the jury received information from two experts in basic and clinical research. The session concluded with an invitation for members to return the following day. On Day 2, the jury received information from social and technological research experts. Then, the deliberation process took place, during which members prioritised the ageing research topics that should be investigated at INGER through voting. At the end of the meeting, the jury completed a satisfaction survey using a Likert scale. The survey assessed their satisfaction with their participation, the clarity of the information provided by the experts, the quality of the activities and materials, the performance of the facilitators, and a final open‐ended question for suggestions or comments. Photography and video recordings were taken throughout the 2‐day meeting.

### 2.2 Processing, Organisation and Analysis of Information

The unit of analysis was the deliberative exercise, which included two stages: the collective and individual deliberation phases, where each person had the opportunity to identify the priority issue(s) from their perspective. All evidence collected during the 2‐day meeting, including jury agreements, group and individual notes, and audio and video recordings, was reviewed to eliminate duplicate responses. However, all original opinions, conclusions, and recommendations were entirely preserved.

After the meeting, two types of reports were produced: an internal evaluation and a final report. A meeting was held with the organising team for the internal review, and written feedback was requested from the group of expert researchers. Additionally, the facilitators' observation notes were included to identify key findings and issues encountered during the meeting. For the final report, all evidence generated during the meeting was transcribed, organised according to the chronological order of the descriptive evidence, and supplemented with photographic documentation. The final report underwent a third‐party validation. In our case, this validation occurred through two phases: (1) verifying that the information in the report aligned with video recordings and (2) requesting the co‐facilitator to review the report, highlighting errors or omissions in the information.

The study was approved by the Research and Ethics Committees of the INGER, with the research project identification: *DI‐PI‐007/2024*. All participants signed an informed consent and were told they could withdraw at any time without explanation.

## 3 Results

We recruited 27 older people who comprised the CJ. Table 1 presents the socio‐demographic survey data. The jury primarily consisted of people aged 60–69, with a majority being women; 74% had at least a completed bachelor's degree, more than half were married or in a union, and 85% had social security. They exhibited low morbidity, and none were reported to have a disability.

**Table 1:** Description of citizens' jury participants.

Variable	Percentage (*n* = 27)
Age	
60–69	55.56%
70–80	37.03%
80 and more	7.41%
Sex	
Men	40.74%
Women	59.26%
Level of education	
No formal education	0%
Primary education completed	0%
Secondary education completed	11.11%
High school or technical degree completed	14.81%
Bachelor's degree completed	55.56%
Master's degree completed	7.41%
Doctoral degree completed	11.11%
Marital status	
Single	11.11%
Married or in a union	51.85%
Widowed	18.52%
Divorced	18.52%
Social security affiliation*	
With	85.19%
Without	14.81%
Chronic diseases**	
0	40.74%
1	40.74%
2 or more	18.52%
Disability	
With	0%
Without	100%

* Social security Mexican institutions: Instituto Mexicano del Seguro Social, IMSS; Instituto de Seguridad y Servicios Sociales de los Trabajadores del Estado, ISSSTE; Petróleos Mexicanos, PEMEX; Secretaría de la Defensa Nacional, SEDENA; and Secretaría de Marina, SEMAR.

** The chronic diseases questioned included type 2 diabetes, hypertension, cardiovascular disease, stroke, cancer, respiratory diseases, and kidney diseases.

### 3.1 Deliberation Process

During the deliberation process, CJ also discussed, as expected, benefits and suggestions for each research area to reach a consensus about research priorities. Tables 2 and 3 present the results of the deliberation stage during the collective and the individual phase, respectively.

**Table 2:** Collective deliberation by research area.

Research area	Benefits	Recommendations
Biomedical research	− It provides simple and individualised practical solutions to improve people's quality of life − It supports clinical and applied research lines	− To define research lines in coordination with other regions in the country − To do basic research in preventive medicine − To disseminate and communicate the research lines among the patient care by the clinical medical unit at INGER
Clinical epidemiologic research	− Generate information to create programs that improve the quality of life of older people − Be able to influence the development of a new health care model at a national level − This study is less expensive, takes less time and is based on biomolecular knowledge	− To increase the dissemination to obtain more participation of old persons − To integrate in a cross‐sectional manner the psychosocial lines with the rest of the research areas
Socio‐demographic research	− Allows scientists to go out into society or receive information from the environment − We recognise that INGER is implementing long‐term research − Takes real data to favourably impact the development of public policies	− Incorporate the gender perspective
Geronto‐technology research	− Helps to reduce the digital divide − Greater development of technologies − Ageism, old age and its link to disability are being eradicated through this study lines − Use existing technological guidelines for the benefit of people and develop our own technologies aimed at the Mexican elderly public	− To develop intergenerational groups that allow technological exchange − Incorporate artificial intelligence − To strengthen the relationship with other institutes, to focus resources on what exists or does not exist (academia, health, institutions, research, etc.)

*Source:* Own elaboration, transcripts of evidence from the meeting.

**Table 3:** Deliberation by personal opinions.

Research carried out at INGER	
Benefits	Recommendations
− To be open to citizen participation − To work on technological advances − Giving voice to older people as protagonists − Committed and loving team − Technological tools − Cutting‐edge ideas − Interaction between areas for the benefit of older people	− Take advantage of what already exists in technology − To include psychology research lines − To disseminate better the achievements of INGER − To strengthen the reduction of the digital divide − Incorporate more artificial intelligence (AI) − More research funds − More teamwork with and among older people − New line of research in the preventive area − More search for new drugs for new diseases − Gender and intercultural perspective

*Source:* Own elaboration, transcripts of evidence from the meeting.

Regarding research priorities in ageing, we organised the information after analysing data from the field notes and audiovisual recordings of the deliberative process. The most relevant points are presented in Table 4.

**Table 4:** Priority research topics defined by the citizens' jury.

Priority ranking	Basic research area	Clinical research area	Socio‐demographic research area	Technological research area
1	Preventive medicine in cognitive, physical and nutritional dimensions	Vision care	Life course research	Digital divide in elders, with a focus on training the older persons in the use of technologies, networks, platforms and applications (Apps)
2	Develop new drugs to more efficiently address common diseases affecting older adults	Mental health, with a focus on emotional care	Gender influence research	Incorporate new technologies in research, like artificial intelligence
3	Alternative medicine	Sexual health	Intergenerational research	Development of elder‐friendly devices
4		Dental health	Intercultural research	Strategies to make accessible technology for older persons
5		Nutritional health	Digital divide in elders	Adapt existing technologies for older adults by improving biometric data collection, enhancing accessibility for those with visual impairments or physical limitations
6		Alternative medicine	Social and self‐inflicted ageism	Enhance security in data collection
7		Palliative care and thanatology	City infrastructure requirements for older people	
8		Health promotion aimed at the elderly	Impact of financial State assistance	
9		Development of interventions to improve the quality of life in older people	Care for the elderly, with a focus on carers' health	
10			Lack of resources in the health system	

Additionally, general recommendations were gathered from the jury. Most of them were repeated across each area, and we have summarised them in Table 5 in non‐specific order.

**Table 5:** General recommendations on ageing research.

1. Create a new investigation area specifically focused on the prevention of diseases
2. Develop an informative strategy to raise awareness about the benefits of ageing
3. Focused on translational research by applying findings from basic research to clinical research. Additionally, integrating social and clinical data into the formulation of public policies
4. Give a voice to older people as protagonists (citizen participation)
5. Increase the compromise of public institutions to address the problems of older people
6. Increase diffusion of research findings to the general population
7. Increase financial resources for ageing research
8. Make more efficient use of scarce resources by leveraging existing technology to provide solutions, rather than repeating research
9. Organise more institutional events targeting older people to include them in research
10. Promote intersectional research by collaborating with public institutions, private organisations, non‐governmental organisations (NGOs) and Patient and Family Advisory Councils (PFACs)

### 3.2 CJ Descriptive Process

During the first day, there was mistrust and resistance to group integration techniques; this situation was overcome almost immediately. During the experts' presentations, the CJ listened, took notes and was reflective. There was always an opportunity for them to express their concerns to each researcher. The group dynamics shifted from mistrust to integration, commitment and critical dialogue at the end of the second day. Both days featured a closing session, during which the group was reflective, grateful and emotional. The participants demonstrated a strong willingness to integrate during the breaks on both days, establishing a close relationship and communicating outside of the CJ process.

One of the greatest challenges was maintaining horizontal relationships among the members. For example, this situation was anticipated in the meeting design, and a group presentation technique was proposed to prioritise the collective. However, at the group's insistence, at the beginning of the second day, they were allowed to introduce themselves individually. Each member began to share their professional experience, highlighting their very high educational levels. The result of this presentation favoured group integration, but it also generated power relations among the participants. One of the members, who called herself a ‘simple housewife’, began to appear very insecure, but the facilitators remained close to her to reassure her and recognise her participation.

Power relations were also observed in the collective deliberations. Some spoke more than others or tried to position issues that the majority did not support. This was the reason why the facilitators gave each participant a voice and vote in the deliberations and made this explicit within the group. They were asked to be supportive of the time allowed for participation in this democratic exercise.

Another situation that arose during the first day was that some of the participants, with extensive professional experience and some with experience managing groups, approached the facilitators to give instructions on how to modify the order of the presentations and to point out behaviours they considered inappropriate on the part of their peers. This was resolved by listening respectfully to the participants, showing interest in their contributions and acknowledging their commitment to the CJ.

At the end of the meeting, the jurors expressed their gratitude for their participation. Throughout the process, they demonstrated great commitment. Some of the most frequent comments were: ‘I want to participate openly, objectively, and honestly in the process’, ‘Thank you for prioritizing older people and their rights’, and ‘My commitment as a citizen juror is to help understand the priorities of older people’. The jurors also expressed that they had never heard of an activity like this.

### 3.3 Satisfaction Survey

The response rate from the satisfaction survey was 85% (23 out of 27 participants). All participants agreed that the main objective of the jury was achieved; 74% were satisfied with the information provided by the experts, and 87% felt that the time was sufficient to answer the questions posed. However, in open responses, some members suggested extending the duration of the CJ due to the large amount of information they would need to process to give an informed verdict, and due to the need for thorough discussions and careful consideration of all factors. Furthermore, all participants expressed interest in participating in future INGER participation activities or events. They also communicated deep gratitude for the social initiative, highlighting that they were pleased with INGER's project to include older people in ageing research.

## 4 Discussion

This CJ was an initial effort to engage older people in a participatory process that had not been previously used with this age group in Mexico. This technique also promotes older people's social participation in the INGER, where the initiative aims to identify research priorities related to ageing. This initiative is particularly relevant given the limited knowledge about the social participation of older people in Latin America. Some studies have shown that older people who actively engage in social activities experience improved subjective well‐being in Chile [30] and a 22% reduction in mortality risk [31]. In Ecuador, efforts have been made to better understand the integration of older men into social activities [32]. However, compared to other world regions, there is still a lack of evidence on the impact of social participation on elders' health in this region [2], especially when compared with younger populations and high‐income countries [15], where older people commonly participate in a wide range of social, cultural, religious, recreational and sports activities [2].

By answering the question ‘What are the priority research topics on ageing that should be investigated at the National Institute of Geriatrics?’, the jurors expressed their main interests across the four research areas developed at INGER. One of the notable findings is that the basic research area received the least attention, with only three topics considered a priority, markedly fewer than in the clinical, socio‐demographic and technological areas. This suggests that older people probably prioritise research fields where they perceive more direct or immediate benefits for their health and well‐being. Currently, we do not have sufficient evidence to confirm a relationship between their health status and their research interests. However, this possible hypothesis could be explored in future research.

Another relevant finding is the interest of older people in technology and its usability. Participants emphasised the importance of learning how to use digital tools and expressed concern about the digital divide, which they believe could be addressed through institutional strategies supported by scientific research. Additionally, two research topics, rarely investigated within the institute, were considered relevant by the jurors: emotional care and sexual health. These topics are often overlooked due to ageist assumptions associated primarily with younger populations [33].

Regarding the general recommendations in Table 5, older people are concerned about financial constraints in research and how these limitations may compromise the continuity of scientific studies. They also emphasised the importance of enhancing the dissemination of findings, promoting prevention‐focused approaches, strengthening intersectoral collaboration and ensuring the active inclusion of older people as participants and contributors to the research process.

Considering the reported results, we assume jurors advocated for more inclusive research agendas with a holistic and comprehensive approach that incorporates a life‐course perspective, gender analysis, intercultural understanding and intergenerational focus. This call should be taken seriously by researchers aiming to produce relevant and socially impactful work.

However, these valuable insights should not be interpreted as mandatory research questions or directives. Rather, these priority topics should be viewed as areas of interest for the older people's community, which researchers are encouraged to review and consider to enhance the social relevance of ageing research. The final report was submitted to the Research Direction at INGER for dissemination among the research institutional community.

By identifying research priorities derived from the CJ, we contribute to knowledge translation (KT), which seeks to apply generated knowledge to benefit society and reduce the gap between *what is known* and *what is being done* [34]. Regarding health ageing research, scientific knowledge has grown significantly over the past century. However, this progress has often been fragmented, costly, poorly integrated, and with limited influence on public health policies [35]. To address these challenges, the WHO developed a KT framework, in which the first step involves understanding the characteristics, needs and contexts of the population targeted by research [36]. In recent decades, growing evidence has demonstrated the importance of involving individuals and communities in the research process, as this approach promotes KT by ensuring that the knowledge generated is relevant to them [37].

This contrasts with the traditional selection of research topics based primarily on researchers' interests, limiting interaction with the populations affected by the issues under study. This disconnection may result in research that fails to address real needs [17, 27, 37]. Consequently, by identifying priority research topics for the target population, researchers could incorporate citizens' perspectives into the definition of research priorities, improving the relevance and impact of their scientific work [26, 34] and promoting more responsive scientific research to the needs of society [38]. Moreover, it is essential to acknowledge that researchers must adapt study designs to include older people in research projects. Historically, this group has been unjustifiably excluded from scientific research due to factors such as research ageism, the additional time needed for informed consent processes or carrying out interventions, and misperceptions about their capacities. This exclusion is especially evident in the common unjustified use of upper‐age limits as exclusion criteria in research studies [39].

### 4.1 Beyond Research: CJ in Public Institutions

This CJ has been an innovative experience that has created a social participation opportunity at the INGER, which benefits the group of participating older people in two fundamental ways: in its empowerment and by strengthening their social skills. The CJ should be considered an important participatory technique to be implemented across social institutions, as social exclusion among older people has been overlooked in public policy. Actual policies that claim to address this issue have mainly focused on unemployment and poverty, without considering all the challenges that older people face. These barriers include both internal and external forms of ageism, low expectations about the value of older people's contributions, and physical and architectural obstacles such as the lack of elevators, ramps and accessible sidewalks. Additional challenges involve poor dissemination of information about participation opportunities, communication difficulties derived from hearing or vision impairments, reduced mobility, and the absence of inclusive participation strategies for those unfamiliar with digital technologies [3, 13, 14].

However, one of the most critical obstacles remains in the public and private institutions that lack social participation opportunities for older people. This is often exacerbated by a general mistrust of institutions. Therefore, institutions have a responsibility to create environments that promote social participation of older people. This requires adapting spaces to the specific needs of this population, actively combating ageism within their structures, encouraging empathy, and transforming internal practices to become more inclusive [14]. This institutional commitment should be sustained over the long term, given the growing number of older people and the need to better understand their needs. Such efforts could contribute to the development of more effective public policies, the creation of age‐friendly institutions, and improved outcomes of interventions. Additionally, its potential benefits should be explored in future research.

Moreover, actions resulting from social participation processes are often perceived as more legitimate, justifiable and feasible compared to those made through traditional top‐down approaches, where decisions are taken exclusively by ‘experts’, frequently leaving behind the voices of citizens [16, 20].

Finally, the socio‐demographic profile of the older participants in this CJ aligns with previous findings, which indicate that older people with higher levels of education and women are more likely to participate in scientific and social initiatives [38]. This may be partly explained by women's higher life expectancy [40, 41, 42], as well as by specific motivations among highly educated older people, such as a genuine interest in the research topic, the desire to enhance the relevance and usefulness of research, the pursuit of personal fulfilment or a sense of purpose, a wish to contribute to society [38] or personal interests like social interaction [39].

Based on the satisfaction survey, we observed a high level of satisfaction among older people regarding the CJ process, with all participants agreeing that the objective had been achieved. However, their responses also suggest that extending the duration of the CJ could allow for a deeper discussion of the topic. This recommendation is reinforced by the participants' strong interest in taking part in future INGER activities or events, as well as their appreciation for the institute for creating meaningful opportunities for social participation. Although this is an important finding regarding social participation in older people, we must recognise certain limitations regarding this strategy. The group was composed mainly of individuals with high levels of education, especially when compared to the general population [43], which could shape the findings by focusing the research topics on technology and digital access. Additionally, the jury does not represent the diversity of the older population in terms of educational attainment. Also, due to the sample method selection, older people who didn't have electronic devices or internet access were excluded from the CJ. Also, logistical barriers, such as difficulties accessing INGER, may have prevented some individuals from participating.

## 5 Conclusion

The CJ is a deliberative strategy that can be used to foster social participation among older people. It also enables their active involvement in diverse public issues, such as, in our case, the identification of research priorities. By engaging older people in this way, we enhance KT by aligning research agendas with topics that matter to this age group. Therefore, we recommend the continued and expanded use of this strategy to support evidence‐informed public policy‐making [44] and to promote the meaningful contributions of older people not only in the health sector but also across cultural, economic, political and other social domains.

## Author Contributions

**Nancy Flores‐Castillo:** methodology, writing – review and editing, formal analysis, data curation, investigation, visualisation. **Carmen García‐Peña:** conceptualisation, investigation, validation, writing – review and editing, project administration, supervision, resources, visualisation. **Sara Torres‐Castro:** investigation, writing – review and editing, validation. **Héctor García‐Hernández:** investigation, conceptualisation, writing – original draft, writing – review and editing, methodology, validation, software, visualisation.

## Ethics Statement

The study was approved by the Research and Ethics Committees of the National Institute of Geriatrics, with the research project identification: *DI‐PI‐007/2024*.

## Consent

All participants provided informed consent before their involvement in the Citizen Jury. They were fully informed about the entire process, including the purpose and structure of a Citizen Jury and the expected outcomes. Also, they were encouraged to ask questions regarding the documents and were informed of their right to withdraw from the study at any time without providing any justification. The informed consent documents were sent to participants 2 weeks before the jury, but were formally signed on the first day of the meeting. These signed consents are stored under the responsibility of the corresponding author and are accessible only to the research team. All procedures followed the Mexican Law on Health Research (https://salud.gob.mx/unidades/cdi/nom/compi/rlgsmis.html).

## Conflicts of Interest

The authors declare no conflicts of interest.

## Data Availability

The data that support the findings of this study are available on request from the corresponding author. The data are not publicly available due to privacy or ethical restrictions.
